# Evaluation of right ventricular function by Doppler tissue imaging of the tricuspid annulus in patients with acquired immune deficiency syndrome

**DOI:** 10.3892/etm.2014.1480

**Published:** 2014-01-08

**Authors:** HAOHUI ZHU, JIANJUN YUAN, CHANGHUA WEI, JIYUN CHEN, YISA WANG

**Affiliations:** Department of Ultrasound Diagnostics, People’s Hospital of Zhengzhou University, Zhengzhou, Henan 450003, P.R. China

**Keywords:** human immunodeficiency virus, acquired immune deficiency syndrome, right ventricular dysfunction, tissue Doppler imaging, echocardiography, tricuspid annulus movement

## Abstract

Chronic heart disease contributes to the mortality of patients with AIDS. Although studies of left ventricular function in patients with acquired immune deficiency syndrome (AIDS) have been conducted, studies of right ventricular function are rare. The present study aimed to characterize the tricuspid annulus movement and evaluate the right ventricular function of patients with AIDS by tissue Doppler imaging. Tissue Doppler echocardiography was performed on 106 patients with AIDS and 64 controls. Tricuspid annulus movements were detected from the apical four-chamber view and the apical right heart two-chamber view. The peak diastolic early period velocity (Ve), peak diastolic later period velocity (Va) and peak systolic velocity (Vs) were measured at the anterior, posterior and lateral walls and also at the interventricular septum. Mean values were calculated, as well as the Tei index of the lateral site. Compared with the values in the control group, the Vs and Va of the AIDS group decreased at all sites with the exception of the lateral wall, whereas the Ve decreased at all sites of the tricuspid annulus (P<0.05). The Tei index was higher in the AIDS group than in the control (P<0.05). The results obtained in the present study show that the function of the right ventricle decreases in patients with AIDS, which is indicative of susceptibility to right ventricular dysfunction.

## Introduction

Acquired immune deficiency syndrome (AIDS) is a serious disease that spread rapidly in the world since 1984. AIDS has spread rapidly in China as well as numerous other developing countries. During the end of the last decade, AIDS spread rapidly through serum sampling in Henan Province, China. Chronic heart disease contributes to the mortality of individuals with AIDS (1,2). Although studies of left ventricular function in patients with AIDS have been conducted (3–6), studies of right ventricular function are rare, thus, the phenomenon remains unclear. For this reason, the present study investigated the characteristics of tricuspid annulus movement and evaluated right ventricular function in patients with AIDS by Doppler tissue imaging (DTI), which is a quantitative method that evaluates ventricular myocardial function, to explore whether the right ventricular function was damaged in patients with AIDS.

## Materials and methods

### Patients

In total, 106 patients with AIDS were enrolled in the study, comprising 46 males (43.40%) and 60 females (56.60%), aged 20–59 years, with a mean age of 41.26±7.47 years. The CD4 counts ranged between 1 and 390 cells/μl, with a mean of 185.09±118.12/μl. All cases were infected by serum sample and were confirmed to be infected with the testing of plasma samples (GE Medical Systems, Horton, Norway) . The infection period was between 4 and 27 years, with a mean of 11.69±4.04 years. The AIDS group was compared with an age- and gender-matched population of 64 normal subjects. No abnormal observations in the control group were observed by physical examination, X-ray, electrocardiography (ECG) and echocardiography. None of the enrollees had a medical history of cardiovascular abnormalities and none of the patients with AIDS had undergone highly active antiretroviral therapy. The study was conducted in accordance with the Declaration of Helsinki and with approval from the Ethics Committee of the People’s Hospital of Zhengzhou University (Zhengzhou, China). Written informed consent was obtained from all participants.

### Methods

Echocardiography was performed in all enrollees using American GE Vivid 3 or 7 ultrasound systems (GE Healthcare, Little Chalfont, UK), with transducer frequencies of 1.7–3.5 and 1.5–4.0 MHz, respectively. Enrollees were positioned in the left-lateral position and had normal respiration. Doppler tissue velocity mode was used and focused at the tricuspid annulus level, with a sample volume between 4 and 5 mm. Care was taken in directing the transducer beam as close as possible to the DTI beam at <20° in selected planes. Tricuspid annulus movements in all subjects were detected from the apical four-chamber view and the apical right heart two-chamber view. The peak diastolic early period velocity (Ve), peak diastolic later period velocity (Va) and peak systolic velocity (Vs) were measured at four sites (anterior, posterior, lateral wall and interventricular septum) and the mean value of these variables, including ‘s’, ‘e’ and ‘a’, were calculated. The DTI spectrum of tricuspid annulus movements includes three waves: ‘s’, movement towards the apical during systole; ‘e’, quick movement towards the roof of the atrium during early diastole; and ‘a’, movement caused by atrium systole during late diastole. At the lateral site of the tricuspid annulus, the interval from the end of the ‘a’ wave to the beginning of the next ‘e’ wave, was recorded as line A and the interval from the beginning to the end of the ‘s’ wave was recorded as line B. The Tei index was calculated using the following formula: (A – B)/B. All parameters were measured three times and mean values were obtained.

### Statistical analysis

Data were analyzed using SPSS version 11.5 (SPSS, Inc., Chicago, IL, USA) and are expressed as mean ± SD. A Student’s t-test was used to compare the mean values between the two groups. P<0.05 was considered to indicate a statistically significant difference.

## Results

**Figure 1:**
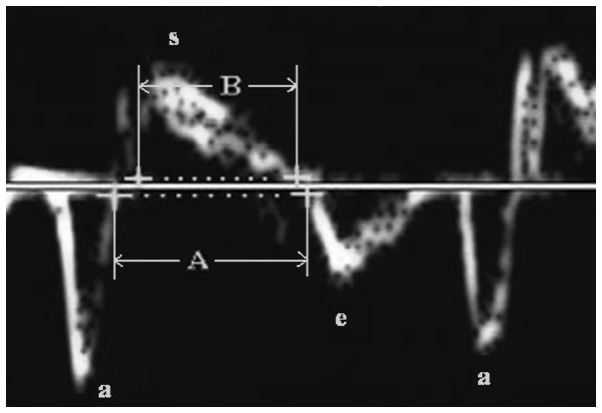
DTI wave of tricuspid annulus movement in a patient with AIDS. Lines A and B were used to calculate the Tei index. DTI, Doppler tissue imaging; AIDS, acquired immune deficiency syndrome.

Fig. 1 shows the ‘s’, ‘e’ and ‘a’ movement waves of the tricuspid annulus in a patient with AIDS. Compared with the values in the control group, the Vs and Va of the AIDS group decreased in all sites with the exception of the lateral wall and the Ve decreased in all sites of the tricuspid annulus (P<0.05). The mean values of Vs, Ve and Va at the four sites of the tricuspid annulus showed a marked reduction in the AIDS group (P<0.05). The Tei index increased significantly in the patients with AIDS (P<0.05). All values are shown in Table I. These results show that the systole and diastole functions of the right ventricle were decreased in the patients with HIV/AIDS, indicating that these individuals are susceptible to right ventricular dysfunction.

## Discussion

Recently, the improved control of opportunistic infection has resulted in a greater awareness of cardiovascular complications in individuals who are HIV positive or have AIDS (7–9). Generally, cardiac complications are asymptomatic and may be masked by symptoms of other complications, which leads to negative results for X-ray and ECG examinations (10,11). This highlights the importance of echocardiography in HIV-associated cardiac dysfunction. Studies on cardiac dysfunction in HIV/AIDS patients frequently investigate the left ventricle (12–15). The ultrasound results included M-mode, pulsed Doppler or strain. DTI is a technique that may be employed to quantitatively study right ventricular function. The technique measures the movements of the tricuspid annulus or right ventricle myocardia, which reflect right ventricular function. In addition, DTI is not affected by morphology, pre-load, respiration, valve area or regurgitant (16). Similarly, the technique is more direct and accurate than pulsed Doppler or the M-mode in assessing right ventricular function by the flow of the tricuspid and pulmonary valves or by the change of right ventricle volume. At present, there are a limited number of studies on the determination of right ventricular function in HIV-positive individuals using DTI (17).

The Tei index is a parameter that reflects the systolic and diastolic function of the heart (18). The results of the present study showed that the DTI values of the tricuspid annulus decreased, while the Tei index increased in patients with AIDS. This indicated that the function of the right ventricle in the AIDS patients was damaged; however, the mechanism is not clear. Left ventricular dysfunction in patients with HIV/AIDS may be caused by a number of factors, including HIV infection, opportunistic infection, autoimmunity, malnutrition, long-term immunodepression, neoplasm and pharmaceuticals (19–23). Right ventricular dysfunction may also be caused by the combined effect of pulmonary diseases, including pulmonary infection and pulmonary microvascular embolism due to embolus formation or drug injection. These diseases increase pulmonary vascular resistance, pulmonary artery hypertension and right ventricular dysfunction. In addition, HIV itself may play a role in the development of right ventricular dysfunction. However, the effects of other factors, including the pathogeny of opportunistic infection, immunomechanisms, pharmaceutical toxicity and neoplasms are unclear and the development of right ventricular dysfunction may be due to multifactorial effects. The present study demonstrated that the right ventricular function of patients with AIDS was damaged, but the mechanism was unclear, and further study is required.

## Figures and Tables

**Table I:** Contrast between the DTI parameters of the AIDS and control groups.

Site	Parameter	AIDS	Control	P-values
Anterior wall	Vs (mm/sec)	11.35±2.20	13.19±2.69	<0.001^a^
	Ve (mm/sec)	12.72±3.06	15.44±3.20	<0.001^a^
	Va (mm/sec)	10.95±3.68	13.16±3.54	0.001^a^
Posterior wall	Vs (mm/sec)	11.23±2.18	12.21±2.50	0.018^a^
	Ve (mm/sec)	12.45±3.43	14.84±3.87	<0.001^a^
	Va (mm/sec)	10.71±3.39	12.34±4.78	0.026^a^
Interventricular septum	Vs (mm/sec)	9.05±1.60	11.45±2.42	<0.001^a^
	Ve (mm/sec)	10.71±1.83	14.85±3.62	<0.001^a^
	Va (mm/sec)	8.26±1.88	10.64±3.11	<0.001^a^
Lateral wall	Vs (mm/sec)	14.11±2.83	14.14±2.64	0.095
	Ve (mm/sec)	12.97±2.38	15.53±3.62	<0.001^a^
	Va (mm/sec)	13.86±4.30	14.70±4.13	0.284
Mean of four sites	S (mm/sec)	11.44±1.45	12.74±2.09	<0.001^a^
	E (mm/sec)	12.21±1.93	15.17±3.05	<0.001^a^
	A (mm/sec)	10.97±2.45	12.71±3.18	0.001^a^
Lateral wall	Tei index	0.41±0.11	0.30±0.16	<0.001^a^

a P<0.05 was considered to indicate a statistically significant difference.

DTI, Doppler tissue imaging; AIDS, acquired immune deficiency syndrome; Vs, peak systolic velocity; Ve, peak diastolic early period velocity; Va, peak diastolic later period velocity.

## References

[1] Lekakis J, Ikonomidis I (2010). Cardiovascular complications of AIDS. Curr Opin Crit Care.

[2] Barbaro G, Silva EF (2009). Cardiovascular complications in the acquired immunodeficiency syndrome. Rev Assoc Med Bras.

[3] Montgomery DE, Puthumana JJ, Fox JM, Ogunyankin KO (2012). Global longitudinal strain aids the detection of non-obstructive coronary artery disease in the resting echocardiogram. Eur Heart J Cardiovasc Imaging.

[4] Ho JN, Yoon HG, Park CS, Kim S, Jun W, Choue R, Lee J (2012). Isothiocyanates ameliorate the symptom of heart dysfunction and mortality in a murine AIDS model by inhibiting apoptosis in the left ventricle. J Med Food.

[5] Chen F, Bhardwaj R, Finkel MS (2012). Diastolic dysfunction following HIV infection. AIDS.

[6] Mondy KE, Gottdiener J, Overton ET, SUN Study Investigators (2011). High prevalence of echocardiographic abnormalities among HIV-infected persons in the era of highly active antiretroviral therapy. Clin Infect Dis.

[7] Fisher SD, Kanda BS, Miller TL, Lipshultz SE (2011). Cardiovascular disease and therapeutic drug-related cardiovascular consequences in HIV-infected patients. Am J Cardiovasc Drugs.

[8] Ogalha C, Luz E, Sampaio E (2011). A randomized, clinical trial to evaluate the impact of regular physical activity on the quality of life, body morphology and metabolic parameters of patients with AIDS in Salvador, Brazil. J Acquir Immune Defic Syndr.

[9] Sims A, Hadigan C (2011). Cardiovascular complications in children with HIV infection. Curr HIV/AIDS Rep.

[10] Miller LH, Coppola JT (2011). Noninvasive assessment of HIV-related coronary artery disease. Curr HIV/AIDS Rep.

[11] Baker JV, Lundgren JD (2011). Cardiovascular implications from untreated human immunodeficiency virus infection. Eur Heart J.

[12] Lipshultz SE, Williams PL, Wilkinson JD, Pedeatric HIV/AIDS Cohort Study (PHACS) (2013). Cardiac status of children infected with human immunodeficiency virus who are receiving long-term combination antiretroviral therapy: results from the Adolescent Master Protocol of the Multicenter Pediatric HIV/AIDS Cohort Study. JAMA Pediatr.

[13] Bajwa AA, Cury JD, Jones L, Shujaat A, Usman F (2012). Echocardiographic findings and their impact on outcomes of critically ill patients with AIDS in the era of HAART. Pulm Med.

[14] Reinsch N, Kahlert P, Esser S (2011). Echocardiographic findings and abnormalities in HIV-infected patients: results from a large, prospective, multicenter HIV-heart study. Am J Cardiovasc Dis.

[15] Blaylock JM, Byers DK, Gibbs BT (2012). Longitudinal assessment of cardiac diastolic function in HIV-infected patients. Int J STD AIDS.

[16] Jones DK, Leemans A (2011). Diffusion tensor imaging. Methods Mol Biol.

[17] Rocha MO, Barbosa FB, Martins MA, do Nunes MC (2012). Patient with chronic Chagas heart disease, hepatosplenic schistosomiasis and acquired immunodeficency syndrome: possible spontaneous resolution of thrombus in the right ventricle. Rev Soc Bras Med Trop.

[18] Tei C, Ling LH, Hodge DO (1995). New index of combined systolic and diastolic myocardial performance: a simple and reproducible measure of cardiac function - a study in normals and dilated cardiomyopathy. J Cardiol.

[19] Fedele F, Bruno N, Mancone M (2011). Cardiovascular risk factors and HIV disease. AIDS Rev.

[20] Choi AI, Vittinghoff E, Deeks SG, Weekley CC, Li Y, Shlipak MG (2011). Cardiovascular risks associated with abacavir and tenofovir exposure in HIV-infected persons. AIDS.

[21] Reinsch N, Neuhaus K, Esser S, German Competence Network Heart Failure; German Competence Network for HIV/AIDS (2012). Are HIV patients undertreated? Cardiovascular risk factors in HIV: results of the HIV-HEART study. Eur J Prev Cardiol.

[22] Vernon LT, Babineau DC, Demko CA (2011). A prospective cohort study of periodotal disease measures and cardiovascular disease markers in HIV-infected adults. AIDS Res Hum Retroviruses.

[23] Farinatti PT, Borges JP, Gomes RD, Lima D, Fleck SJ (2010). Effects of supervised exercise program on the physical fitness and immunological function of HIV-infected patients. J Sports Med Phys Fitness.

